# Sustained Effectiveness of Upadacitinib in Moderate-to-Severe Atopic Dermatitis: A 48-Week Real-World Study

**DOI:** 10.3390/ph17040519

**Published:** 2024-04-18

**Authors:** Teppei Hagino, Risa Hamada, Mai Yoshida, Hidehisa Saeki, Eita Fujimoto, Naoko Kanda

**Affiliations:** 1 Department of Dermatology, Nippon Medical School Chiba Hokusoh Hospital, Inzai 270-1694, Japan; 2 Department of Dermatology, Nippon Medical School, Tokyo 113-8602, Japan; h-risa@nms.ac.jp (R.H.); m-mai@nms.ac.jp (M.Y.); h-saeki@nms.ac.jp (H.S.); 3 Fujimoto Dermatology Clinic, Funabashi 274-0063, Japan; doctor@fujimoto-derma.com

**Keywords:** atopic dermatitis, upadacitinib, Janus kinase, long-term, real-world

## Abstract

Clinical trials and real-world studies have shown the effectiveness of upadacitinib for treating rash and pruritus in patients with atopic dermatitis (AD). This study aimed to determine whether the early reduction in rash or pruritus at week 12 of upadacitinib treatment could be maintained at later treatment stages. This retrospective study involved 227 and 73 patients with moderate-to-severe AD treated with 15 and 30 mg upadacitinib daily, respectively. The eczema area and severity index (EASI) scores, peak pruritus numerical rating scale (PP-NRS), and investigator’s global assessment (IGA) were analyzed. At week 12, patients were divided into achievers and non-achievers of EASI 75, 90, 100, absolute EASI ≤ 2, IGA0/1, PP-NRS4, or absolute PP-NRS ≤ 1. Achievement rates for each endpoint were assessed at later time points (weeks 24, 36, and 48) in both groups. Week 12 achievers largely maintained their endpoint achievements until week 48, regardless of dosage (15 mg or 30 mg). Week 12 non-achievers saw an increasing achievement rate of EASI 75 until week 48. The initial reduction in rash and pruritus at week 12 persisted until week 48 with upadacitinib treatment, suggesting potential benefits for patients requiring prolonged treatment despite not achieving EASI 75 at week 12.

## 1. Introduction

Atopic dermatitis (AD) is a chronic inflammatory skin disease characterized by a type 2-skewed immune response, pruritus, and impairment of the skin barrier [1,2]. Previous studies have shown that the development of AD is related to specific cytokines, such as interleukin (IL)-4, IL-5, IL-13, IL-22, IL-31, and thymic stromal lymphopoietin, which intracellularly signal via the Janus kinase (JAK)/signal transducer and activator of transcription pathways [3]. The Janus kinase inhibitors upadacitinib, baricitinib, and abrocitinib have been approved as systemic treatments for AD. Clinical trials and real-world studies have shown the efficacy and safety of upadacitinib for moderate-to-severe AD [4,5,6,7,8,9,10,11]. A post hoc analysis of the Phase III JADE COMPARE trial showed that patients who achieved ≥4-point improvement on the peak pruritus numerical rating scale (PP-NRS) at week 2 of abrocitinib treatment achieved higher rates of the eczema area and severity index (EASI) 75, 90, and investigator‘s global assessment (IGA) 0/1 at week 12 compared to non-achievers [12]. Patients who achieved absolute PP-NRS ≤ 1 at week 2 attained a higher rate of EASI 100 at weeks 12 and 24 compared to non-achievers [13]. In the BREEZE-AD3 study, patients with AD receiving baricitinib who achieved IGA ≤ 2 at week 16 maintained high rates of EASI 75 and IGA 0/1 until week 68 [14]. These results suggest that early improvement in pruritus or rash may predict strong therapeutic outcomes in the later stages of treatment with baricitinib and abrocitinib. However, it remains unclear whether the early reduction in rash or pruritus is sustained at later stages of upadacitinib treatment. 

This study aimed to investigate whether the early improvement in rash or pruritus at week 12 could be maintained until week 48 of upadacitinib treatment in real-world clinical practice. We also investigated whether patients with an insufficient response to upadacitinib at week 12 could achieve greater therapeutic effects at later stages of treatment.

## 2. Results

### 2.1. Demographic and Baseline Characteristics

Of the 300 patients with AD in this study, 227 and 73 were treated with 15 mg and 30 mg of upadacitinib daily, respectively. The baseline demographics of patients in the two treatment groups are presented in Table 1. The proportion of pretreatment with dupilumab and baricitinib (4 mg) was higher in the 30 mg group than in the 15 mg group, indicating more refractory symptoms of AD in the former group. Baseline EASI, IGA, and PP-NRS scores were lower in the 30 mg group than in the 15 mg group, which may reflect the effects of systemic pretreatments in the former group. The number of week 12 achievers and non-achievers of individual endpoints was longitudinally assessed throughout the 48 weeks of treatment (Table 2 and Table 3). Table 2 presents the longitudinal analysis of achievement rates for EASI 75, EASI 90, and EASI 100 responses, while Table 3 shows the outcomes for EASI ≤ 2, IGA 0/1, PP-NRS 4, and PP-NRS ≤ 1.

### 2.2. The Transition of Achievement Rates of EASI 75 at the Later Stages of Upadacitinib Treatment

**Figure 1:**
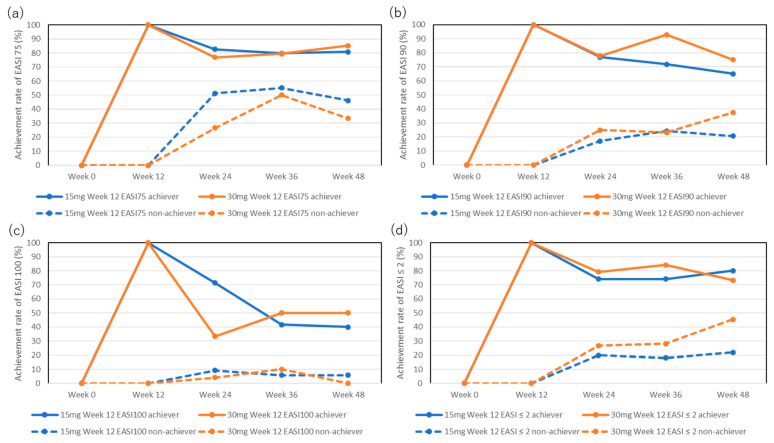
Achievement rates for EASI 75 (**a**), EASI 90 (**b**), EASI 100 (**c**), and absolute EASI ≤ 2 (**d**) in week 12 achievers or non-achievers during treatment with upadacitinib (15 mg or 30 mg daily).

The achievement rate for EASI 75 in week 12 achievers was mostly maintained in both the 15 mg and 30 mg groups (Figure 1a): 82.7%, 79.7%, and 80.6% in the 15 mg group and 76.9%, 79.4%, and 85.2% in the 30 mg group at weeks 24, 36, and 48, respectively.

The achievement rate for EASI 75 in week 12 non-achievers increased at later stages: 51.2%, 55.2%, and 46.2% in the 15 mg group and 26.7%, 50%, and 33.3% in the 30 mg group at weeks 24, 36, and 48, respectively, although there were no statistically significant differences in the achievement rates compared to week 12 (0%).

### 2.3. The Transition of Achievement Rates of EASI 90 at the Later Stages of Upadacitinib Treatment

The achievement rates for EASI 90 in week 12 achievers in the 15 mg group slightly decreased at the later stages, although this was not statistically significant compared to week 12 (100%): 76.9%, 71.9%, and 65% at weeks 24, 36, and 48, respectively (Figure 1b). The achievement rate for EASI 90 in week 12 achievers in the 30 mg group was mostly maintained: 77.8%, 92.9%, and 75% at weeks 24, 36, and 48, respectively.

The achievement rate for EASI 90 in week 12 non-achievers in the 15 mg group slightly increased during the later stages, although this was not statistically significant compared to week 12 (0%): 17.2%, 24.2%, and 20.8% at weeks 24, 36, and 48, respectively. The achievement rate for EASI 90 in week 12 non-achievers in the 30 mg group also slightly increased during the later stages without statistically significant differences compared to week 12 (0%): 25%, 23.3%, and 37.5% at weeks 24, 36, and 48, respectively.

### 2.4. The Transition of Achievement Rates of EASI 100 at the Later Stages of Upadacitinib Treatment

The achievement rates for EASI 100 in week 12 achievers decreased during the later stages of treatment in both the 15 mg and 30 mg groups, although without statistically significant differences compared to week 12 (100%): 71.4%, 41.7%, and 40% in the 15 mg group, and 33.3%, 50%, and 50% in the 30 mg group at weeks 24, 36, and 48, respectively (Figure 1c).

The achievement rate of EASI 100 in week 12 non-achievers was low and did not significantly increase during the later stages: 9.2%, 5.8%, and 5.9% in the 15 mg group and 4.2%, 10%, and 0% in the 30 mg group at weeks 24, 36, and 48, respectively.

### 2.5. The Transition of Achievement Rates of EASI ≤ 2 at the Later Stages of Upadacitinib Treatment

The achievement rates for EASI ≤ 2 in week 12 achievers were mostly maintained during the later stages in both groups (Figure 1d): 74.1%, 74.1%, and 80% in the 15 mg group and 79.2%, 84.2%, and 73.3% in the 30 mg group at weeks 24, 36, and 48, respectively.

The achievement rates for EASI ≤ 2 in week 12 non-achievers in the 15 mg group slightly increased during the later stages, although this was not statistically significant compared to week 12 (0%): 20%, 18.1%, and 22% at weeks 24, 36, and 48, respectively. The achievement rate for absolute EASI ≤ 2 in week 12 non-achievers in the 30 mg group increased, although this was not statistically significant, compared to week 12 (0%): 26.7%, 28%, and 45.5% at weeks 24, 36, and 48, respectively.

### 2.6. The Transition of Achievement Rates of IGA 0/1 at Later Stage of Upadacitinib Treatment

**Figure 2:**
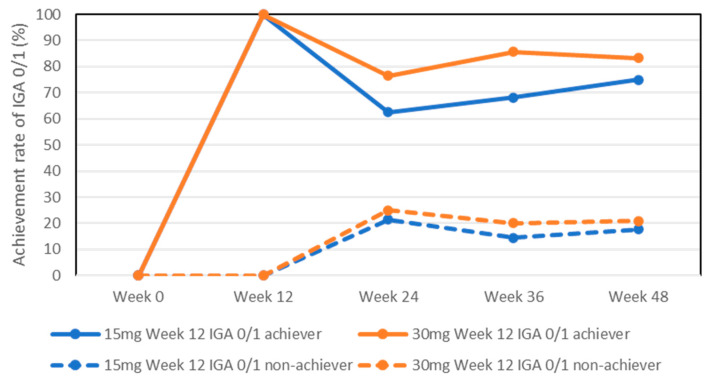
Achievement rates for IGA 0/1 in week 12 achievers or non-achievers during treatment with upadacitinib (15 mg and 30 mg daily).

The achievement rates for IGA0/1 in week 12 achievers in the 15 mg group slightly decreased at later stages, although without statistically significant differences compared to week 12 (100%): 62.5%, 68.2%, and 75% at weeks 24, 36, and 48, respectively (Figure 2). The achievement rates for IGA0/1 in week 12 achievers in the 30 mg group were mostly maintained: 76.5%, 85.7%, and 83.3% at weeks 24, 36, and 48, respectively.

The achievement rates for IGA0/1 in week 12 non-achievers in both the 15 mg and 30 mg groups slightly increased at later stages, although without statistically significant differences compared to week 12 (0%): 21.5%, 14.5%, and 17.7% in the 15 mg group, and 25%, 20%, and 20.8% in the 30 mg group at weeks 24, 36, and 48, respectively.

### 2.7. The Transition of Achievement Rates of PP-NRS 4 at the Later Stages of Upadacitinib Treatment

**Figure 3:**
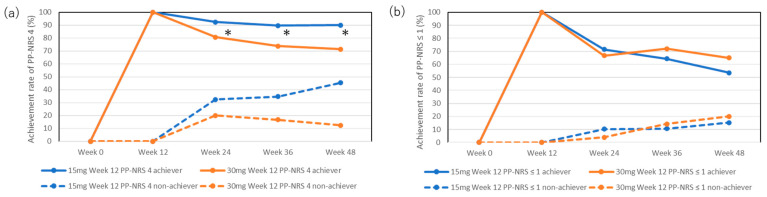
Achievement rates for PP-NRS 4 (**a**) or PP-NRS ≤ 1 (**b**) in week 12 achievers or non-achievers during treatment with upadacitinib (15 mg or 30 mg daily). * *p* < 0.05, week 12 achievers of PP-NRS 4 in the 15 mg group versus those in the 30 mg group, analyzed using Fisher’s exact test.

The achievement rates for PP-NRS 4 in week 12 achievers in the 15 mg group were mostly maintained during the later stages: 92.4%, 89.7%, and 90% at weeks 24, 36, and 48, respectively (Figure 3a). The achievement rates for PP-NRS 4 in week 12 achievers in the 30 mg group slightly decreased at the later stages, although this was not statistically significant compared with week 12 (100%): 80.8%, 73.9%, and 71.4% at weeks 24, 36, and 48, respectively. The achievement rates for PP-NRS 4 were significantly higher in week 12 achievers in the 15 mg group than those in the 30 mg group at weeks 24, 36, and 48.

The achievement rates for PP-NRS 4 in week 12 non-achievers in the 15 mg group increased during the later stages, although this was not statistically significant compared with week 12 (0%): 32.4%, 34.6%, and 45.4% at weeks 24, 36, and 48, respectively. The achievement rates for week 12 non-achievers in the 30 mg group slightly increased during the later stages, although this was not statistically significant compared to week 12 (0%): 20%, 16.7%, and 12.5%.

### 2.8. The Transition of Achievement Rates of Absolute PP-NRS ≤ 1 at the Later Stages of Upadacitinib Treatment

The achievement rates for PP-NRS ≤ 1 in week 12 achievers in both the 15 mg and 30 mg groups decreased during the later stages, although this was not statistically significant compared to week 12 (100%): 71.4%, 64.3%, and 53.6% in the 15 mg group and 66.7%, 72%, and 65% in the 30 mg group at weeks 24, 36, and 48, respectively (Figure 3b).

The achievement rates for PP-NRS ≤ 1 in week 12 non-achievers in both the 15 mg and 30 mg groups were low and did not significantly increase during the later stages: 10.3%, 10.7%, and 15.2% in the 15 mg group, and 4%, 14.3%, and 20% in the 30 mg group at weeks 24, 36, and 48, respectively.

### 2.9. Adjusted Transition of Achievement Rates of Clinical Indexes at the Later Stages of Upadacitinib (30 mg) Treatment

Including patients previously treated with upadacitinib (15 mg) could potentially show unclear results due to the lack of response to upadacitinib (30 mg) treatment. Therefore, an analysis was conducted after excluding patients who had been treated with upadacitinib (15 mg) from the upadacitinib 30 mg group. New figures were created for the achievement rates of EASI 75, EASI 90, EASI 100, and EASI ≤ 2 (Supplemental Figure S1a–d), the achievement rate of IGA 0/1 (Supplemental Figure S2), and PP-NRS ≤ 1, PP-NRS 4 (Supplemental Figure S3a,b), similar to existing ones. Essentially, the results for the upadacitinib 30 mg group, after excluding patients with a history of upadacitinib (15 mg) treatment, showed almost similar outcomes, suggesting that prior treatment with upadacitinib (15 mg) might not have influenced the results.

## 3. Discussion

In this study, the achievement rates for EASI 75, 90, EASI ≤ 2, IGA0/1, PP-NRS 4, or PP-NRS ≤ 1 in week 12 achievers were consistent until week 48 of upadacitinib treatment. Although the achievement rate for EASI 100 in week 12 achievers decreased at later stages, the differences in frequency compared to week 12 were not statistically significant, possibly because of the decrease in the number of patients in later phases. Consistent with this study, a previous clinical trial of the JAK 1/2 inhibitor baricitinib reported long-term maintenance of therapeutic effects in early responders/partial responders who achieved IGA ≤ 2 at week 16 [14]. Specifically, in the baricitinib (4 mg) treatment group, the early responders/partial responders maintained the achievement rate of validated IGA-AD 0/1: 45.7% or 47.1% at week 16 or 68, respectively, although that of EASI 75 slightly decreased from 70% at week 16 to 55.7% at week 68. Similarly, in the baricitinib 2 mg treatment group, early responders/partial responders maintained achievement rates of validated IGA-AD 0/1 (46.3% or 59.3%) and EASI 75 (74.1% or 81.5%) at weeks 16 or 68, respectively. The results of that study indicated the long-term maintenance of early responses to JAK inhibitors in the treatment of AD.

Our previous real-world study showed that the achievement of IGA 0/1 at week 12 may be predicted by lower baseline EASI and higher age in the 15 mg upadacitinib treatment group and by lower immunoglobulin E and LDH in the 30 mg treatment group [15]. This indicates that patients with the above background may achieve IGA0/1 at week 12 and maintain a good response in the later stages of upadacitinib treatment at the respective doses.

In this study, the achievement rates of EASI 75 in week 12 non-achievers in both the upadacitinib 15 mg and 30 mg groups increased at later stages of treatment until week 48, although the differences were not statistically significant compared with week 12. These results indicate that slow responders may exist latently among early non-responders to upadacitinib treatment. Relating to our findings, a post hoc analysis of the dupilumab open-label extension study [16] revealed that patients who did not achieve EASI 75 or IGA 0/1 at week 16 in SOLO 1 or 2 studies had high achievement rates at week 100: 91% for EASI 75 and 45% (biweekly treatment) or 49% (monthly treatment) for IGA 0/1. In our present study, the reason why some non-achievers of EASI 75 at week 12 benefited from prolonged treatment with upadacitinib remains unknown. However, it may be related to the patient’s phenotypes/endotypes, genetic factors, or medication adherence levels. Our current findings indicate that some early non-responders at week 12 may benefit from continued upadacitinib treatment, implying a need for a longer evaluation period to fully assess the responsiveness to upadacitinib. Although determining the assessment time point is challenging, our results suggest that monitoring effects for up to a year might be reasonable. Patients who achieved EASI 75 at a later stage without early response may exhibit higher baseline EASI scores and younger age in the 15 mg treatment group or higher baseline IgE and LDH in the 30 mg treatment group, as analogized from our previous real-world data [15]. Alternatively, slow responders may mainly present with lichenification, whose response to upadacitinib is delayed compared to the other clinical signs, excoriation, erythema, or edema/papulation [11].

In this study, we observed that week 12 non-achievers of EASI 100, IGA 0/1, or PP-NRS ≤ 1 did not show a trend towards increased achievement rates of these endpoints at later stages of treatment until week 48. Achieving these endpoints suggests a complete or near-complete resolution of rash or pruritus, indicating that the potential to attain these stringent endpoints might be limited to early responders by week 12.

It is a very critical issue to choose between the continuation of upadacitinib treatment or switching to another treatment for patients who did not achieve EASI 75 at week 12. Because nearly half of the patients may potentially achieve EASI 75 in the later phase of treatment, physicians may continue treatment. However, this continuation may have a psychological impact on patients with AD, subjecting them to a potential insufficient response for a prolonged period. Identifying predictive factors for long-term effectiveness or slow responders could aid in choosing appropriate treatments for early non-responders by week 12.

Week 12 non-achievers of PP-NRS 4 in the 15 mg group showed higher achievement rates of PP-NRS 4 at the late stage compared to the 30 mg group, although the difference was not statistically significant (Figure 3a). This is possible because patients in the 15 mg group had higher baseline PP-NRS values and might have more room to reduce their PP-NRS than those in the 30 mg group (Table 1). Furthermore, the maintenance rate of PP-NRS 4 in week 12 achievers was significantly higher in the 15 mg group than in the 30 mg group (Figure 3a). These results were rather unexpected and may be because of bias owing to the much smaller number of week 12 achievers in the 30 mg group than that in the 15 mg group (Table 2) and/or because week 12 achievers in the 15 mg group may have a higher potential to sustain the response to upadacitinib on pruritus compared to those in the 30 mg group. The results indicate that the therapeutic effects of upadacitinib on pruritus may not consistently align with those on rash and may not always be dose-dependent.

Reports on the long-term effectiveness and safety of upadacitinib in managing AD in real-world clinical studies are increasing, illustrating the sustained therapeutic potential of the drug. This is highlighted in studies from Italy, such as those by Chiricozzi A et al. 2023 and Gargiulo et al. 2023, as well as the systematic review by Ibba L et al. [17] and others [8,10,18,19,20]. Chiricozzi A et al. detailed the outcomes for 146 patients with moderate-to-severe AD, most of whom (87.0%) received upadacitinib as monotherapy [8]. With 80.8% of these patients on a daily dose of 30 mg, significant clinical improvements were observed at week 16, and achievement rates of EASI 75, EASI 90, and EASI 100 were 78.2%, 47.6%, and 28.2%, respectively, at 16 weeks. At week 48, the achievement rates of EASI 75, EASI 90, and EASI 100 reached 87.6%, 69.1%, and 44.3%, respectively. The study underscored the consistent effectiveness of upadacitinib throughout the observation period. Gargiulo L et al.’s retrospective analysis of 71 patients demonstrated high achievement rates of IGA 0/1 (90.9%), EASI 75 (87.9%), EASI 90 (75.8%), and EASI 100 (57.6%) after one year, with significant symptom relief reported [10]. The effectiveness of upadacitinib was robust, regardless of previous dupilumab exposure, and the study reported no serious AEs. Ibba L et al.’s systematic review further confirms the long-term effectiveness and safety of upadacitinib for severe AD in real-world studies [17]. Through these comparative analyses, the vital role of upadacitinib in the AD treatment landscape is reinforced, endorsing its application as a primary treatment option for achieving long-term disease control.

This study had some limitations. First, the observation period of 48 weeks may be insufficient to fully assess the long-term effectiveness of upadacitinib treatment for AD. Future studies with longer treatment durations are necessary to provide a more comprehensive understanding of the long-term effects of this drug. Second, there was an imbalance in the number of patients between the upadacitinib 15 mg and 30 mg groups, indicating the need for further investigation with a more balanced proportion of participants. Thirdly, this study predicts the maintenance of effectiveness at week 48 based on the classification of subjects into achievers and non-achievers of various clinical indexes at week 12. While we identified baseline patient characteristics that reflect short-term effectiveness in our previous studies with upadacitinib (15 mg and 30 mg) at week 12, future research should focus on identifying baseline characteristics that could predict long-term effectiveness based on the clinical indexes evaluated in this study (achievement rate of EASI 75, 90, 100, IGA 0/1, EASI ≤ 2).

## 4. Materials and Methods

### 4.1. Study Design and Data Collection

This retrospective study was conducted from August 2021 to November 2023 and involved 300 Japanese patients (aged ≥ 12 years) with moderate to severe AD. These patients, diagnosed with AD based on the Japanese Guidelines for Atopic Dermatitis 2021, were identified as having moderate to severe AD with EASI ≥ 16 or head-and-neck EASI ≥ 2.4. All patients received daily oral upadacitinib (15 mg or 30 mg) combined with moderate to strong topical corticosteroids twice daily. Before upadacitinib treatment, data were collected on the patient’s age, sex, body mass index, disease duration, history of bronchial asthma, allergic conjunctivitis, allergic rhinitis, and previous treatment with dupilumab, upadacitinib (15 mg), or baricitinib (4 mg). This study was conducted in accordance with the Declaration of Helsinki (2004) and approved by the Ethics Committee of Nippon Medical School Chiba Hokusoh Hospital. Written informed consent was obtained from all the patients.

### 4.2. Outcomes of Effectiveness

The EASI, PP-NRS, and IGA scores were analyzed before and after updacitinib treatment. At week 12, patients were divided into achievers and non-achievers of EASI 75, 90, or 100 (at least a 75%, 90%, or 100% reduction from baseline EASI, respectively), absolute EASI ≤ 2, IGA 0/1 (IGA scores of 0 (clear) or 1 (almost clear)), PP-NRS4 (PP-NRS reduction ≥ 4 points among patients with baseline PP-NRS ≥ 4-point), or absolute PP-NRS ≤ 1 (Table 2). The achievement rate of each endpoint was analyzed at later time points (weeks 24, 36, and 48) in week 12 achievers and non-achievers.

### 4.3. Statistical Analysis

Results were expressed as medians and interquartile ranges for nonparametrically distributed variables. Differences in frequencies were assessed using Fisher’s exact test. Differences between the two groups were analyzed using the Mann–Whitney U test for variables with a nonparametric distribution. Statistical significance was set at *p* < 0.05. In cases of missing data, the affected patients were excluded from the analysis to ensure data integrity and accuracy. All statistical analyses were conducted using EZR software (version 1.55) (Saitama Medical Center, Jichi Medical University).

## 5. Conclusions

The reduction in rash and pruritus achieved at week 12 was maintained until week 48 of upadacitinib treatment. The achievement rate for EASI 75 in week 12 non-achievers increased until week 48 of treatment at both the 15 mg and 30 mg doses. These results indicate that a subset of non-achievers of EASI 75 at week 12 may benefit from prolonged treatment.

## Figures and Tables

**Table 1:** Baseline demographics and disease characteristics of patients with atopic dermatitis treated with upadacitinib.

	Total Population (*n* = 300)	15 mg (*n* = 227)	30 mg (*n* = 73)	*p* (15 mg versus 30 mg)
Male sex, *n* (%)	216 (72.0)	162 (71.4)	54 (74.0)	0.765
Age (years) ^a^	37.0 [18.0–51.0]	35.5 [16.0–51.0]	40.0 [31–48]	0.327
Body mass index (kg/m^2^) ^a^	22.7 [15.6–25.9]	22.2 [20.1–25.0]	23.6 [21.5–27.2]	0.0133 *
Disease duration (years) ^a^	30.0 [15.0–44.0]	28.0 [13.0–44.0]	33 [22–43]	0.114
Presence of allergic conjunctivitis	46 (15.3)	34 (15.0)	12 (16.4)	0.852
Presence of allergic rhinitis	99 (33)	70 (30.8)	29 (39.7)	0.198
Presence of bronchial asthma	94 (31.3)	65 (28.6)	29 (39.7)	0.0829
Pretreatment, *n* (%)				
Previous dupilumab	24 (8.0)	12 (5.3)	12 (16.4)	<0.01 **
Previous upadacitinib (15 mg)	32 (10.7)	NA	32 (43.8)	NA
Previous baricitinib (4 mg)	33 (11.0)	6 (2.6)	27 (37.0)	<0.01 **
Clinical indices				
EASI ^a^	20.3 [14.4–30.0]	23.8 [17.2–32.0]	12.5 [8.5–19.4]	<0.01 **
IGA, *n* (%)				
Mild (score of 2)	51 (17.0)	23 (10.1)	28 (38.4)	<0.01 **
Moderate (score of 3)	153 (51.0)	120 (52.9)	33 (45.2)	
Severe (score of 4)	96 (32.0)	84 (37.0)	12 (16.4)	
PP-NRS ^a^	8.0 [6.0–9.0]	8 [7–9.5]	6 [3–8.0]	<0.01 **

^a^ Data provided as the median [interquartile range]. * Statistically significant at *p* < 0.05, ** *p* < 0.01 by Fisher’s exact test or Mann–Whitney U test. EASI, eczema area and severity index; IGA, investigator’s global assessment; PP-NRS, peak pruritus numerical rating scale; NA, not applicable.

**Table 2:** The number of week 12 achievers and non-achievers of EASI 75, 90, and 100 throughout 48 weeks of upadacitinib treatment.

Outcome	Time Point	Week 12 Achievers in 15 mg Group (%)	Week 12 Achievers in 30 mg Group (%)	*p*	Week 12 Non-Achievers in 15 mg Group (%)	Week 12 Non-Achievers in 30 mg Group (%)	*p*
EASI 75	Week 12	129/182 (70.9)	45/64 (70.3)	0.351	53/182 (29.1)	19/64 (29.7)	1
	Week 24	107/145 (73.8)	34/54 (63)	0.173	38/145 (26.2)	20/54 (37)	0.515
	Week 36	72/99 (72.7)	32/44 (72.7)	0.247	27/99 (27.2)	12/44 (31.8)	0.0606
	Week 48	51/74 (68.9)	26/36 66.7)	0.139	23/74 (31.1)	10/36 (27.8)	0.0606
EASI 90	Week 12	69/182 (37.9)	19/64 (29.7)	1	113/182 (62.1)	45/64 (70.3)	0.289
	Week 24	56/145 (38.6)	23/54 (42.6)	1	89/145 (6.1)	31/54 (57.4)	0.153
	Week 36	40/99 (40.4)	20/44 (45.5)	0.143	59/99 (59.6)	24/44 (54.5)	0.463
	Week 48	25/74 (33.8)	18/36 (50)	0.703	49/74 (66.2)	18/36 (50)	0.0942
EASI 100	Week 12	26/182 (14.4)	6/64 (9.4)	1	156/182 (85.7)	58/64 (90.6)	0.274
	Week 24	22/145 (15.2)	4/54 (7.4)	0.161	123/145 (84.8)	50/54 (92.6)	0.182
	Week 36	11/99 (11.1)	6/44 (13.6)	1	88/99 (88.9)	38/44 (86.4)	0.344
	Week 48	7/74 (9.5)	1/36 (2.8)	1	67/74 (90.5)	35/36 (97.2)	0.086

EASI, eczema area and severity index.

**Table 3:** The number of week 12 achievers and non-achievers of EASI ≤ 2, IGA 0/1, PP-NRS 4, and PP-NRS ≤ 1 throughout 48 weeks of upadacitinib treatment.

Outcome	Time Point	Week 12 Achievers in 15 mg Group (%)	Week 12 Achievers in 30 mg Group (%)	*p*	Week 12 Non-Achievers in 15 mg Group (%)	Week 12 Non-Achievers in 30 mg Group (%)	*p*
EASI ≤ 2	Week 12	75/186 (40.3)	29/64 (45.3)	1	111/186 (59.7)	35/64 (54.7)	0.245
	Week 24	59/149 (40.7)	27/54 (50)	0.778	90/149 (60.4)	27/54 (50)	0.171
	Week 36	34/100 (34)	23/44 (52.3)	0.488	66/100 (66)	21/44 (47.7)	0.173
	Week 48	49/75 (65.3)	21/37 (56.8)	1	26/75 (34.7)	16/37 (43.2)	0.268
IGA 0/1	Week 12	48/175 (27.4)	19/63 (30.2)	1	127/175 (72.6)	44/63 (69.8)	0.262
	Week 24	43/139 (30.9)	22/53 (41.5)	0.36	96/139 (69.1)	31/53 (58.5)	0.248
	Week 36	27/99 (27.3)	18/44 (40.9)	0.432	72/99 (72.7)	26/44 (59.1)	0.275
	Week 48	21/75 (28)	21/36 (58.3)	1	54/75 (72)	15/36 (41.7)	0.38
PP-NRS 4	Week 12	121/168 (72)	29/48 (60.4)	0.199	47/168 (28)	19/48 (39.6)	1
	Week 24	97/129 (75.2)	24/41 (58.5)	0.0347 *	32/129 (24.8)	17/41 (41.5)	0.506
	Week 36	71/95 (74.7)	19/35 (54.3)	0.0471 *	24/95 (40.7)	16/35 (45.7)	0.444
	Week 48	56/73 (76.7)	16/29 (55.2)	0.0401 *	17/73 (23.3)	13/29 (44.8)	0.199
PP-NRS ≤ 1	Week 12	76/179 (42.5)	30/62 (48.4)	1	103/179 (57.5)	32/62 (51.6)	0.425
	Week 24	48/134 (35.8)	19/52 (36.5)	0.611	86/134 (64.2)	33/52 (6.4)	0.25
	Week 36	34/99 (34.3)	21/46 (45.7)	0.593	65/99 (65.7)	25/46 (54.3)	0.474
	Week 48	23/75 (30.7)	16/35 (45.7)	0.392	52/75 (69.3)	19/35 (54.3)	0.294

EASI, eczema area and severity index; IGA, investigator’s global assessment; PP-NRS, peak pruritus numerical rating scale. * Statistically significant at *p* < 0.05 by Fisher’s exact test.

## Data Availability

Data are contained within the article and Supplementary Materials.

## Supplementary Materials

The following supporting information can be downloaded at: https://www.mdpi.com/article/10.3390/ph17040519/s1, Supplemental Figure S1: Achievement rates for EASI 75 (a), EASI 90 (b), EASI 100 (c), and absolute EASI ≤ 2 (d) in week 12 achievers or non-achievers during treatment with upadacitinib 15 mg or 30 mg daily, excluding patients with a history of treatment with upadacitinib 15 mg. Supplemental Figure S2: Achievement rates for IGA 0/1 in week 12 achievers or non-achievers during treatment with upadacitinib 15 mg and 30 mg daily, excluding patients with a history of treatment with upadacitinib 15 mg. Supplemental Figure S3: Achievement rates for PP-NRS 4 (a) or PP-NRS ≤ 1 (b) in week 12 achievers or non-achievers during treatment with upadacitinib (15 mg or 30 mg daily), excluding patients with a history of treatment with upadacitinib 15 mg.
