# Supplementary material for: Sustained Effectiveness of Upadacitinib in Moderate-to-Severe Atopic Dermatitis: A 48-Week Real-World Study
Source: Pharmaceuticals (Basel). 2024 Apr 18;17(4):519. doi: 10.3390/ph17040519 (PMC11054057; doi:10.3390/ph17040519)
Supplement: Supplementary file 1 [file pharmaceuticals-17-00519-s001.zip › pharmaceuticals-2943918-supplementary.pdf]

Supplemental material

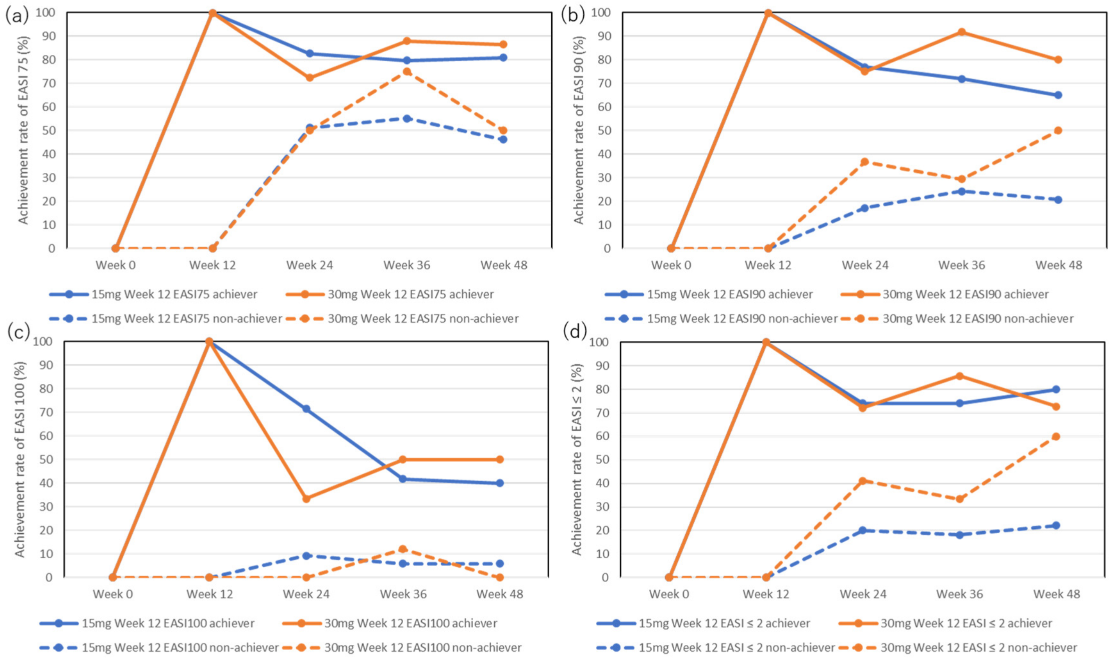

Supplemental Figure S1. Achievement rates for EASI 75 (a), EASI 90 (b), EASI 100 (c), and absolute EASI ≤ 2 (d) in week 12 achievers or non-achievers during treatment with upadacitinib 15 mg or 30 mg daily, excluding patients with a history of treatment with upadacitinib 15 mg.

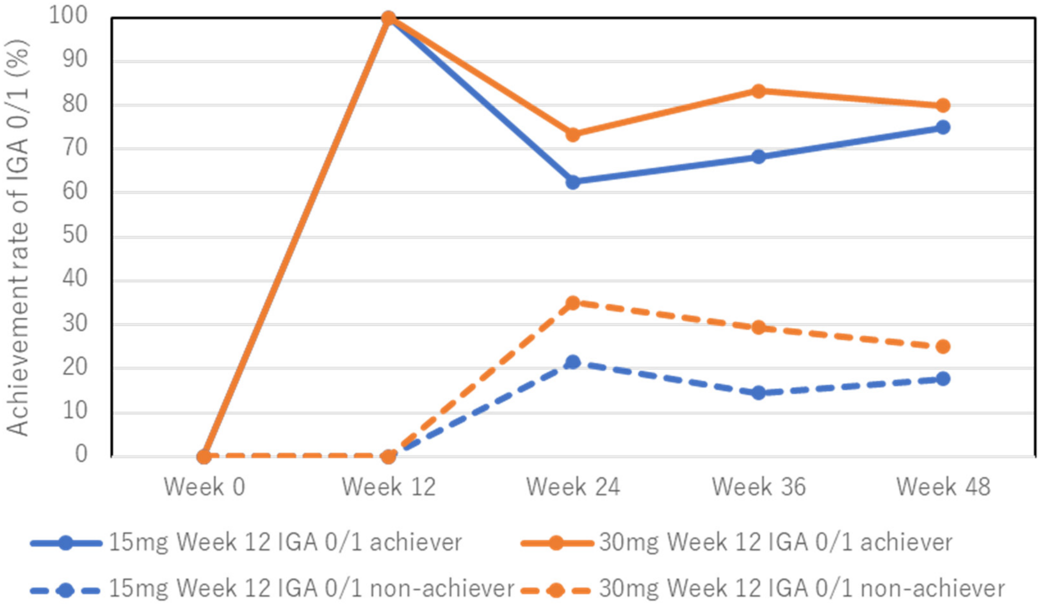

Supplemental Figure S2. Achievement rates for IGA 0/1 in week 12 achievers or non-achievers during treatment with upadacitinib 15 mg and 30 mg daily, excluding patients with a history of treatment with upadacitinib 15 mg.

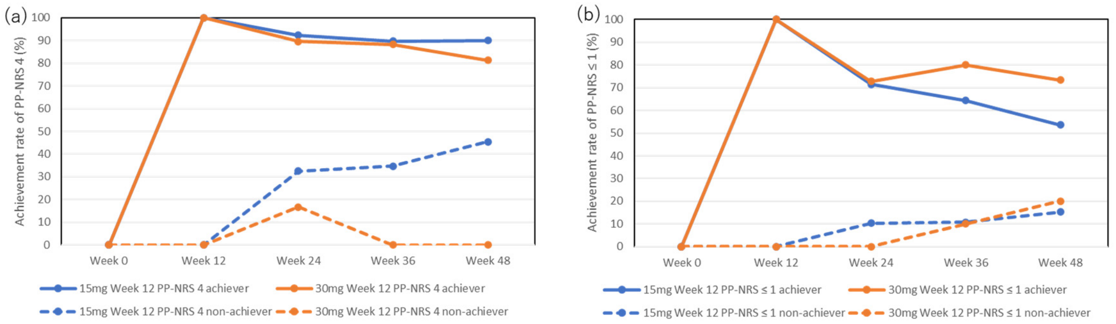

Supplemental Figure S3. Achievement rates for PP-NRS 4 (a) or PP-NRS ≤ 1 (b) in week 12 achievers or non-achievers during treatment with upadacitinib (15 mg or 30 mg daily), excluding patients with a history of treatment with upadacitinib 15 mg.
